# Application of the Buccal Micronucleus Cytome Assay for Genotoxicity Detection in Dogs

**DOI:** 10.3390/ani15030382

**Published:** 2025-01-28

**Authors:** Bruna Filipa Tavares da Costa, Alexandra Teixeira, Joana C. Prata, Daniel Pérez-Mongiovi

**Affiliations:** 1 Sciences Department, University Institute of Health Sciences—CESPU, 4585-116 Gandra, Portugal; 2 Associate Laboratory i4HB—Institute for Health and Bioeconomy, University Institute of Health Sciences—CESPU, 4585-116 Gandra, Portugal; alexandra.teixeira@iucs.cespu.pt (A.T.); joanacorreiaprata@gmail.com (J.C.P.); 3 UCIBIO—Applied Molecular Biosciences Unit, Forensics and Biomedical Sciences Research Laboratory, University Institute of Health Sciences (1H-TOXRUN, IUCS-CESPU), 4585-116 Gandra, Portugal

**Keywords:** genomic damage, exfoliated buccal cells, saliva, biomarker, animal welfare

## Abstract

In Europe, there is a growing concern for animal welfare, encompassing both their rights and health. Consequently, identifying biomarkers that predict serious pathological conditions has become crucial in veterinary medicine. The Buccal Micronucleus Cytome (BMCyt) assay is a minimally invasive method that uses biomarkers to evaluate DNA damage and chromosomal instability, using exfoliated buccal cells. A rising frequency of anomalies, such as micronuclei formation, strongly indicates an elevated risk of cancer, neurodegenerative diseases, or accelerated aging, potentially originating from exposure to genotoxins and cytotoxins. This method has been validated in humans, but very little research has been conducted on animals. This work aims to provide a detailed description of an optimized method for collecting buccal exfoliated cells in dogs and to characterize a biomarker related to genomic damage using optical and fluorescent microscopy. Samples from dogs in breeding kennels, including pregnant animals, were tested for chromosomal instability. By following procedures similar to those used in humans, we were able to detect and count major nuclear abnormalities. The percentage of micronuclei was higher compared to other studies. Technical aspects, such as avoiding artifacts and ensuring prior training of the operator, must be taken into account. This work validated the BMCyt method for collecting and processing samples in dogs, potentially enhancing the understanding of micronuclei as biomarkers for pre-pathological states in canines.

## Simple Summary

Evaluating health and welfare is important for improving veterinary care. One promising method is the Buccal Micronucleus Cytome (BMCyt) assay. This method involves assessing DNA damage in cells collected painlessly from the inside of an animal’s mouth as a marker of disease. Although widely used in humans, BMCyt has rarely been studied in animals. In this work, we optimized and described the use of the BMCyt method in dogs, testing samples from a breeding kennel. Dogs presented genetic damage markers similar to those in humans but at a different frequency. Technical challenges, like improving sample collection and processing, were addressed. Our findings support the potential development of BMCyt assays in dogs as biomarkers of disease.

## 1. Introduction

Animal welfare is an increasing concern in democratic societies, emphasizing both the rights and health of animals [1,2]. Animals have been recognized by the European Union as sentient beings since 2007 [3]. Acknowledging this, the EU adopted a strategy to enhance the welfare of animals used for scientific purposes through Directive No. 2010/63/EU [4]. New rules have also been published regarding the welfare requirements of dogs and cats in shelters, pet shops, and breeding establishments [1]. Companion animals are increasingly seen as family members [5], leading to a higher demand for high-quality veterinary care and the emergence of new diagnostic techniques [6]. However, costs are often the limiting factor for optimal veterinary care [7]. Using biomarkers in veterinary medicine can enable low-cost diagnostics and aid in preventive care by allowing early detection of homeostasis disruptions, and therefore significantly contributing to animal welfare.

A biomarker is a measurable indicator of a specific biological state, usually associated with a disease’s risk, presence, severity, or prognosis [8]. Micronuclei (MN) are abnormal chromosomal structures frequently utilized in humans to assess chromosome instability and the DNA-damaging effects of environmental and endogenous genotoxins [9,10]. The origin of MN can be attributed to clastogenic effects, involving structural chromosome alterations, or aneugenic effects, resulting in numerical chromosome alterations [11]. One method for measuring MN as a biomarker involves using buccal epithelial cells. This minimally invasive method uses micronuclei to evaluate DNA, utilizing exfoliated buccal cells from the internal mucosa of the cheeks. The buccal mucosa is the primary barrier for inhalation and ingestion, making it a high-risk site for exposure to genotoxic agents that enter the body through the aerodigestive tract [12].

More recently, the Buccal Micronucleus Cytome (BMCyt) assay has been proposed. This method extends beyond micronuclei to include the scoring of various buccal cell types presenting nuclear anomalies, offering a comprehensive evaluation of biomarkers for DNA damage, cell death, and cytokinetic defects or arrest [10,12,13].

The BMCyt assay has been utilized in human studies related to occupational exposure, degenerative diseases such as cancer, and lifestyle factors, like drug consumption [10]. However, its application in veterinary medicine remains limited. To our knowledge, few studies have addressed the use of BMCyt in animals [14,15,16,17]. One study found higher genomic damage in purebred dogs than in mixed-breed dogs, attributing this to the high level of inbreeding [16]. Another study found higher genomic damage in cats and dogs living in shelters, compared to home environments, attributing it to higher stress levels [15]. Although literature remains scarce, Appendix A Table A1. summarizes the essence of these studies where the BMCyt assay was tested on various animals. Thus, BMCyt has significant potential as a biomarker for evaluating overall health status. However, BMCyt widespread use in veterinary medicine requires further investigation of methodologies and clarification of basal levels.

Recognizing micronuclei and other abnormalities during cell analysis can be challenging, as subtle details can often be missed. Micronuclei (MN) are small rounded bodies visible in the cytoplasm of cells [9]. They originate from acentric or entire chromatid/chromosome fragments that are left behind during anaphase of mitotic division and are not incorporated into the main nucleus during telophase [13,18]. In contrast, they are surrounded by the nuclear membrane and resemble the structure of the daughter nucleus, although they are much smaller in size [19]. Most factors that give rise to MN are well-known and described in the literature [13,18,20,21,22,23,24,25,26,27]. Other cytogenetic abnormalities, such as binucleated (BN) cells (cells with two nuclei instead of one), can be observed, potentially indicating defects in cytokinesis. Other relevant DNA damage indicators are nuclear buds (NBUDs), which are nuclei with an apparent sharp constriction at one end, where narrow nucleo-plasmic bridges and nuclear buttons are observable [21]. The mechanism leading to NBUDS formation is not well known but it may be related to the elimination of amplified DNA or DNA repair [13,19]. Finally, beyond cells with abnormal nuclei, normal basal cells are typically analyzed to assess proliferative potential [21]. Death of proliferative cells can be caused by various chemical, physical, or biological factors. However, genotoxins do not necessarily kill cells but only damage their genetic material. Although effective DNA repair mechanisms exist, they sometimes fail, leading to the fixation of damage if the affected cell survives. If the damaged cell divides, this damage can be transferred to subsequent generations [26], and these aberrant nuclei are observable in buccal exfoliated cells.

Other types of micronucleus assays such as lymphocyte cytokinesis-block micronucleus (CBMN) cytome, mammalian erythrocyte micronucleus (MEM), or buccal mucosa micronucleus (BMN), have been used for many years [10], CBMN being the best-validated ex vivo assay. BMCyt assay has recently gained increased interest due to its distinct advantages [26]. It is possible to argue that MN in exfoliated oral epithelial cells represents a preferential target site for early genotoxic events induced by carcinogens [18]. This specific micronucleus assay has minimal invasiveness, low cost, ease of performance, and ease of accessing cell samples from the oral cavity compared to tissues such as blood [18,26,28]. This is important in a veterinary context, where rapid, low-impact, and cost-effective diagnostic methods are less abundant than those available for humans. Nevertheless, the BMCyt assay requires thorough testing in animals to evaluate its technical feasibility and overall usefulness.

This study aims to describe and optimize a protocol for collecting and preparing exfoliated buccal cells from dogs, as BMCyt is used for human cells, to assess chromosomal abnormalities, particularly micronuclei, as biomarkers of genotoxicity. Adaptations were introduced to previous studies in animals [14,15,16,17] mainly by the use of both brightfield and fluorescence illumination microscopy as a confirmatory technique. With this purpose, breeding female kennel dogs (including pregnant dogs) of closely related breeds, and environmentally controlled, were selected for application of the technique.

## 2. Material and Methods

### 2.1. Ethical Considerations

This study was carried out following Directive 2010/63/EU (Protection of animals used for scientific purposes) of the European Parliament and was approved by the Ethics Committee of the University Institute of Health Sciences (CE/IUCS/CESPU-04/22). The procedures performed were non-invasive, painless, and did not induce long-term effects on the animals. The breeding kennel owner was informed of the procedures performed on the dogs and signed a free and informed consent form, thereby obtaining consent and authorization from the breeder for the use of the dogs in scientific research.

### 2.2. Animals and Buccal Cell Harvesting

Four purebred French Bulldog dogs and two purebred Pug dogs aged 1 to 4 years, belonging to a single breeder, were the object of study in this work. Only female dogs were used. At the time of sample collection, two out of the six dogs were in an intermediate period of pregnancy, while the remaining four were in a resting period. The sample size was determined by the number of animals in the same kennel, in order to reduce environmental variability, and was deemed suitable for the optimization of the method. Due to the sample size, no statistical analysis was conducted besides descriptive statistics. All female dogs were healthy according to routine health examinations carried out by veterinarians and with vaccinations up-to-date. They were typically fed with commercial diets and housed in a single kennel, which offered both internal and external spaces suitable for their needs. During the entire study, these animals were always handled and managed by the same operator and, after sample collection, they were treated with rewards including dog treats. The dogs had access to food and water prior to the sample collection.

**Figure 1:**
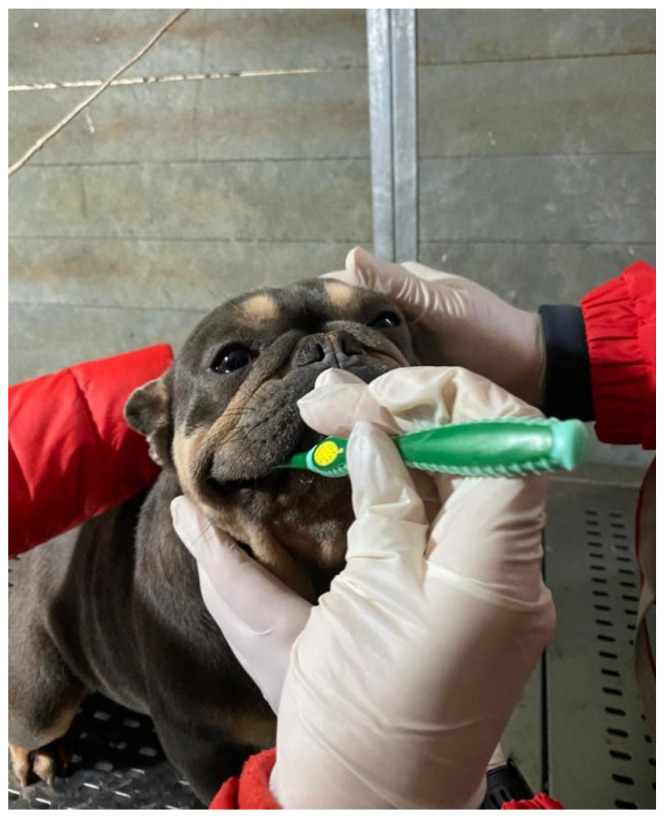
Buccal cell harvesting from a female French Bulldog.

Following the protocol of Thomas and colleagues (2009) [13], exfoliated cells from the oral mucosa were collected using a new medium-bristle toothbrush. The toothbrush was inserted into the oral cavity of the dogs, and the inner lining of both cheeks and along the palatoglossal arch on each side was gently scraped with the brush for at least 15 s (Figure 1).

**Figure 2:**
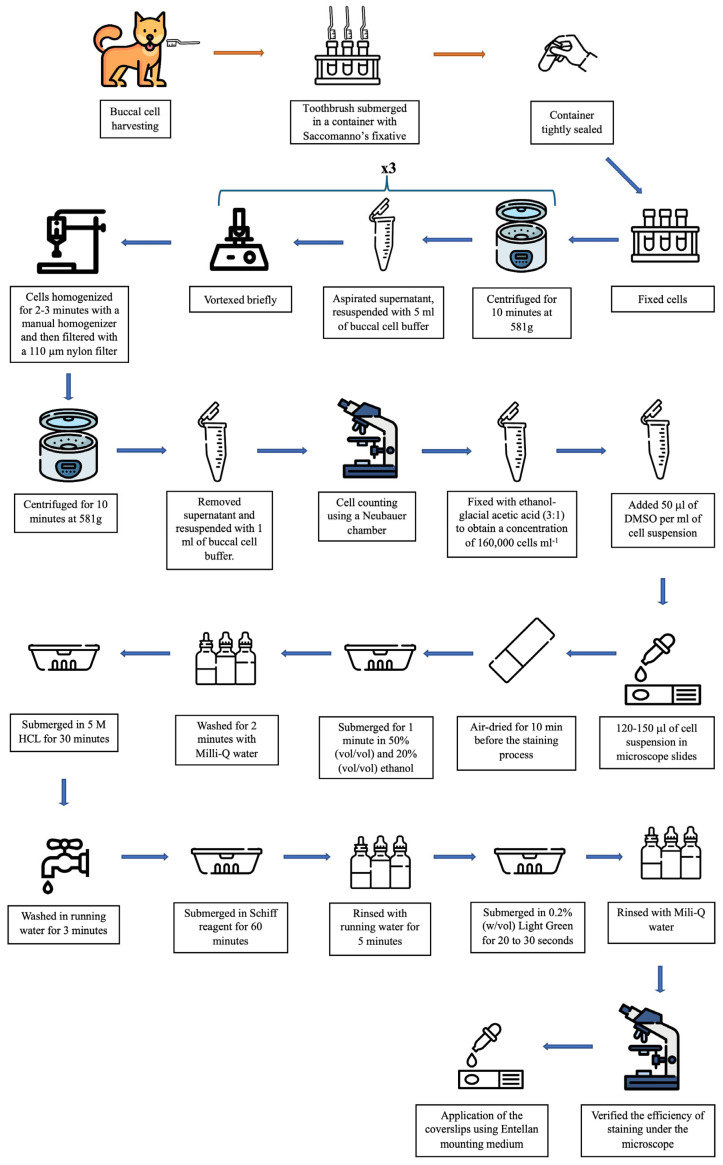
Schematic representation of the complete experimental procedure for collecting and preparing buccal cells for observation.

After this collection, the toothbrush head was submerged and rotated for 30 s in a container containing 10 mL of Saccomanno’s fixative (Ethanol: cat.no. 4146052, Carlo Herba reagents, Milan, Italy; Polyethylene glycol: cat.no. 8.17006, Merck, Darmstadt, Germany); the cells were retained in the solution and the brush was properly discarded. The container was tightly sealed, pre-venting from leakage during transportation to the laboratory. This solution containing the cells can be stored at 4 °C for up to 7 days before preparing slides. Figure 2 shows the schematic representation of the entire experimental procedure until the slides were obtained for microscope observation.

### 2.3. BMCyt Assay

The cells were prepared according to the protocol established by Thomas et al. (2009) [13], with minimal modifications (Figure 2). Firstly, fixed cells were transferred to centrifuge tubes. Cells were centrifuged (Haraeus megafuge 16, Thermo Fisher Scientific, Osterode am Harz, Germany) for 10 min at 581 g at room temperature. The supernatant was aspirated leaving approximately 1 mL of cell suspension and resuspended with 5 mL of buccal cell buffer (Tris base, cat.no. B01601, NZYtech, Lisbon, Portugal; EDTA, cat.no. 131659.1211, Panreac, Darmstadt, Castellar del Vallès, Barcelona, Spain; sodium chloride, Panreac, cat.no. 131659.1211) and vortexed briefly. The buffer is used to help inactivate endogenous DNAses present in the oral cavity and to remove bacteria and cellular debris that can complicate scoring. This process was repeated 3 times to remove impurities, as the dogs did not swish their mouth with water prior to collection and did not undergo regular oral hygiene practices.

Secondly, the solution was homogenized for 2 to 3 min with a 2 mL manual tissue homogenizer (model FY3C194351MQJSO4B7, Scicalife, Shenzhen, China), thus increasing the number of individual cells in suspension. To remove large aggregates of unseparated cells, they were filtered through a 110 µm nylon filter (UPC733063148463, Tong Gu, Tonggu, China). The cells were centrifuged (Spectrafuge 24D, Labnet International, Inc., Edison, NJ, USA) for 10 min at 581 g at room temperature. After removing the supernatant, the cells were then resuspended in 1 mL of buccal cell buffer.

Cell preparation on microscope slides was performed manually. The cells obtained were fixed using the necessary volume of ethanol/acetic acid (3:1) (acetic acid, Panreac, cat.no. 131008.1212) to achieve a concentration of 160,000 cells mL^−1^. A further 50 μL of DMSO (cat.no. 154938, Sigma-Aldrich, Steimheim, Germany) per mL of cell suspension was used to aid in cell disaggregation and to obtain slide preparations with clearly separated cells. A cell suspension of 120–150 μL was placed on clean, dry, properly labeled microscope slides and allowed to air dry for 10 min before staining.

Finally, the microscope slides with the fixed cells were submerged for 1 min each in Coplin flasks containing 50% (*vol*/*vol*) and 20% (*vol*/*vol*) ethanol. Cells were washed for 2 min with Milli-Q water. The slides were then placed in a Coplin flask containing 5 M HCL (cat.no. 30721-12, Honeywell/Fluka, Steimheim, Germany) for 30 min and then washed in running water for 3 min. A negative control was performed only the first time, to verify the efficacy of the 5M HCL treatment.

The slides were drained, not allowed to dry, and placed in a Coplin flask, this time containing the Schiff reagent (Sigma-Aldrich, cat.no. 3952016), for 60 min in the dark at room temperature to stain the nuclei with fluorescence. This was then rinsed with running water for 5 min. For cytoplasm staining, slides were submerged in Coplin flasks containing 0.2% (*w*/*vol*) Light Green (Merck, cat.no. 115941) for 20 to 30 s and rinsed without delay with Mili-Q water. Afterward, to remove any residual moisture, the slides were placed face down on filter paper, not applying any pressure so as not to dislodge the cells.

The slides were placed on a slide tray and allowed to dry for about 10 to 15 min. The efficiency of staining the slides was verified under the microscope at a magnification of ×10 and ×40. The slides were allowed to dry completely over the next day before applying the coverslips. To apply the coverslips, Entellan mounting medium (Merck, cat.no. 1.07961.0500) was used: a large drop was placed on the coverslip, which was then carefully positioned onto the slide. The coverslip was pressed gently to help spread the medium and the creation of air bubbles was avoided. The slides were allowed to dry overnight at room temperature and were covered to prevent dust from settling on them and to avoid light. For each animal (n = 6), two slides were prepared following this protocol.

### 2.4. Microscopes and Scoring Method

For observation of the cells, a confocal microscope, Axio Observer Z.1 SD microscope (Carl Zeiss, Oberkochen, Germany), coupled to an AxioCam MR3, and with the Plan Apochromatic 63×/NA 1.4 objective, was used with the Plan Apochromatic 40×/NA 1.4 objective. In addition, a fluorescent microscope, Nikon TE 2000-U microscope (Nikon, Amsterdam, The Netherlands), was coupled to an DXM1200F digital camera, and Nikon ACT-1 software version v2.x (Melville, NY, USA), was used with the objective ×60 to take pictures. The Feulgen staining technique allows observation under brightfield, as well as fluorescence microscopy using a far-red filter. In brightfield, the staining of the whole cells can be observed: the nucleus is stained in magenta while the cytoplasm is stained in green. Cells viewed under FL DsRed filter fluorescence (emission wavelength range of 580 to 620 nm) have the nucleus and MN-stained red. It was determined that scoring the cells using both brightfield and fluorescence microscopy would be preferable in order to minimize the occurrence of false positives for the abnormal nuclei.

A minimum of 1000 cells were counted per sample, and the frequency of different cell types was determined in the samples from each dog. The criteria for scoring the various types of oral mucosal cells were intended to classify cells into categories that distinguish “normal” cells from cells considered “abnormal”, based on cytological features that indicate normal or abnormal nuclear morphology. The cell counts were supervised by a second operator. In this work, the oral cells were observed and scored, following the criteria of Tolbert et al. (1992) and Thomas et al. (2009) [13,29]. The summarized description of buccal mucosa cells can be found in Table 1.

## 3. Results

The protocol was successfully applied to dog cells with a few small adjustments: an additional wash and spin were performed after the fixative to better clean cellular and non-cellular debris; the cell concentration was increased before placing the cells on the slides to ensure a greater number of cells for counting, and the exposure time with the green light dye was extended by a few seconds for improved visualization of the cell cytoplasm.

**Figure 3:**
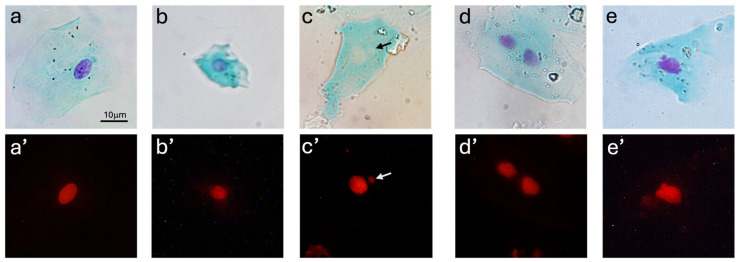
Images of the different cell types and abnormalities observed using brightfield (**a**–**e**) and fluorescent microscope (**a’**–**e’**) scored in the BMCyt assay: (**a**,**a’**) Normal cell, (**b**,**b’**) Basal cell, (**c**,**c’**) Cell with at least one micronucleus, (**d**,**d’**) Binucleated cell, (**e**,**e’**) Cell with a nuclear bud. Notice in (**c**,**c’**) the loss of the purple coloration of chromatin in brightfield, but not in far-red fluorescence (arrows). The scale bar applies to all figures.

In each case, 1000 cells were counted and assessed for various nuclear abnormalities, including MN, BN, and NBUDs, along with evaluation of cell proliferation in the basal cells. Figure 3 illustrates examples of the cell abnormalities observed in the samples taken from the dogs. Chromosomal abnormalities can be small and occasionally mistaken for non-specific staining, sometimes showing a decrease in color intensity (Figure 3).

**Figure 4:**
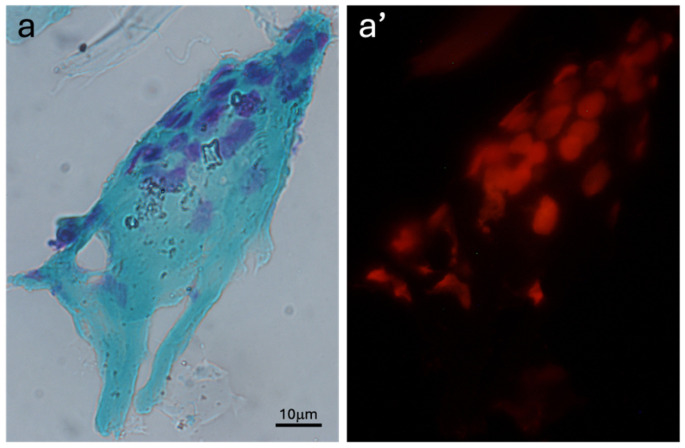
Image showing an example of cell aggregation observed using a brightfield (**a**) and a fluorescent illumination (**a’**). The scale bar applies to all figures.

Occasionally, cell aggregates appeared, which were excluded from the counting (Figure 4). These aggregates may contain cellular overlaps, and some staining, likely corresponding to nuclear fragments or non-specific structures.

The total scored cells from the six dogs are presented in Table 2. Cases 3 and 4 involved pregnant dogs who gave birth a week after the cell collection. They exhibited average values of abnormalities similar to those of the non-pregnant dogs. Comparisons between breeds also seem to reveal a relatively similar number of abnormalities. In case 1, one of the micro-nucleated cells contained five micronuclei. Overall, 0.73% of the buccal cells in the animals studied exhibited abnormalities.

## 4. Discussion

Our study suggests the validity of the BMcyt assay for studying genomic damage in dog buccal cells. A total of 6000 cells from female dogs were counted, revealing a relevant number of abnormal nuclei. We also found it useful during counting to perform a qualitative comparison of the microscopic images using two different illumination techniques. We analyzed samples using Feulgen reaction, enabling the simultaneous use of both fluorescence and brightfield illumination. Double-checking cells enabled us to confirm abnormalities that are challenging to distinguish, such as NBUD or MN, or sometimes poorly stained nuclei (see Figure 3). While fluorescence observation provided rapid visualization of target structures, it occasionally posed challenges due to artifacts (non-specific staining) or residual DNA remnants, which could be verified with brightfield illumination, improving the reliability of results. In other studies, where the BMcyt assay was employed in animals, Giemsa dye or Feulgen reaction was used to stain the nuclei, but only brightfield illumination was applied [14,15,16,17,30]. According to Thomas et al. (2009) [13], Giemsa reagent is known to produce artifacts more frequently. In our understanding, fluorescent illumination can also reveal some non-specific staining of artifacts. Therefore, we recommend double-checking using both brightfield and fluorescence illumination for accuracy.

Our results from the BMcyt assay in dogs clearly show higher MN values compared to human cells [13,21] (Table 3). If we consider that healthy humans have an average of one MN per 1000 cells, the breeding dogs in this study show a threefold increase. However, BN cells are significantly less frequent in dog cells, suggesting a lower incidence of cytokinesis defects. The study conducted by Thomas et al. (2009) [13] used the BMcyt assay on healthy young and elderly humans. Interestingly, they found an average of 27.4 basal cells per 1000 cells in young individuals and 93.53 basal cells per 1000 cells in elderly individuals (Table 3). The present study in dogs revealed a higher number of basal cells, comparable to those found in elderly humans. A technical artifact explanation could be that brushing in the same spot repeatedly during sampling may increase the number of basal cells from the less differentiated basal layer [13]. However, this is unlikely to have occurred during collection, as the brushing was thorough and conducted on both cheeks. Nevertheless, other possibilities exist: the number of basal cells may reflect the mitotic activity and regenerative capacity of the oral mucosa. This number increases in response to cell damage, such as the loss of surface mucosal cells, as suggested by the consumption of hot beverages in humans [31]. It is known that increased mitotic activity can lead to replication errors, resulting in genetic damage. Therefore, it is not surprising to observe a correlation between higher basal cell values and an increase in DNA abnormalities in elderly humans, just as it would not be surprising to find such a correlation in dogs. This correlation needs to be explored in dogs and other animals to establish a baseline for buccal cell proliferation.

In Santovito’s study [15], differences were found between shelter dogs and dogs in family environments, with an increased number of MN in shelter dogs. These dogs were mixed-breed and no correlation was found between the increase in MN and either the sex or age of the dogs. This, along with other behavioral tests, suggests that the environment influences the increase in stress-associated genomic damage in oral cells.

We might consider whether the breeds of dogs or the inbreeding process itself influences the higher number of micro-nucleated cells observed in our results. In a more recent study conducted by Santovito et al. [17], it is suggested that, in pure-bred dogs, inbreeding may increase the levels of genomic damage. The possible stressful effect of the shelter was avoided by using dogs that lived in a family environment. Still, a clear increase in MN level was observed (Table 3). Again, no correlation was found between the increase in MN and either the sex or age of the dogs. The dogs in our study presented more micro-nucleated cells than those observed in pure-bred dogs in Santovito’s study [17], but similar to those observed in shelter dogs [15]. Although the breeding conditions in the present study were considered good, we cannot rule out the possibility that the kennel environment and the dogs’ breeding status could have an additive effect, potentially increasing genomic damage. The combined effect of stress-associated and inbreeding-associated genomic damage in buccal cells should be thoroughly investigated. Moreover, the present sample only included brachycephalic breeds, which are predisposed to multiple diseases [32]. Therefore, future studies should also assess MN levels of different dog breeds.

A different assay measuring MN levels, the lymphocyte CBMN assay, has proven to be predictive of disease risk in prospective studies related to cancer risk, cardiovascular disease mortality, and pregnancy complications in humans [10]. In dogs and other animals, MN levels in lymphocytes were also measured for different purposes; for instance, MN levels in lymphocytes were used as a predictive variable for tumor response during chemotherapy [33], the genotoxic effect of cadmium consumption in dogs [34] or the assessment of baseline levels of DNA damage in marine mammals [35].

The BMcyt assay used in the present study for genomic damage detection has advantages over other biomarkers with the same goal, such as the lymphocyte CBMN assay. It is reliable, with a cost-effective laboratory procedure, and is minimally invasive for the population submitted to the assay [10,15]. The BMcyt assay has been proven to be useful as a biomarker in occupational exposure of workers to genotoxins, potential carcinogens, and radiation [10,13]. Additionally, lifestyle factors in humans, such as drug consumption, smoking, chewing betel and areca nuts, and consuming khat leaves, lead to increased buccal MN frequencies, indicating a risk of developing degenerative diseases. Buccal cells also hold the potential for identifying inherited genomic instability, such as Bloom’s Syndrome [36].

Thus, the association of MN in buccal cells with certain diseases appears to be as robust as that of MN in lymphocytes, since higher levels of MN are found in both types of cells in diagnosed cancers [10]. The potential human clinical application of the BMcyt assay has been claimed as a test to identify patients with a high risk of degenerative diseases. However, no studies have been published so far demonstrating that an increased MN frequency in buccal cells is prospectively associated with a higher risk of disease. More research is needed in both humans and other animals to demonstrate predictive effectiveness and facilitate clinical application.

The use of the BNcyt assay could become an alternative biomarker in cases in which blood collection is complicated. Analysis based on blood collection in small animals is limited by their body size, with the standard being the safe collection of 1% of lean body weight, which is challenging in pediatric patients, smaller breeds, and exotic animals [37,38]. In these animals, sample volumes might be insufficient to run traditional tests based on hematology and biochemistry.

The BNcyt assay has its limitations, but they are comparatively minor when compared against other diagnostic methods. While it may be considered laborious (due to the multiple steps) and time-consuming (executing the whole procedure and counting takes approximately 1–2 days to complete), it is faster when compared to cytologic and histological analysis [26]. Fluorescence MN analysis may require a more specialized type of microscope, but in general it uses widely available equipment and reagents. An observer with expertise in distinguishing different cell typologies and stringent criteria for observers to raise concordance in scoring are required. However, this training is not as technically complex as instruction in histology/pathology [10]. An important consideration for cell collection timing is determining the optimal moment for harvesting buccal cells. The formation of micronuclei (MN) in the basal layer takes approximately one to three weeks before cells with MN appear in the surface layers of the buccal mucosa in humans [13,39]. Furthermore, exposure to genotoxic events can be either acute or chronic. Therefore, it is crucial to consider both the duration and intensity of the exposure to select appropriate collection times and ensure an accurate micronucleus frequency count. Regarding the specific animal, there may also be limitations when dealing with aggressive individuals; in these cases, as with traditional diagnostic methods, sedation may be necessary to collect the sample.

Looking ahead, automating the scoring of MN in exfoliated buccal cells appears to be a feasible and promising approach. Reliability can be enhanced by minimizing bias associated with varying nuclear abnormalities recognition by different analysts or mistakes provoked by analyst fatigue [40]. Encouraging results have been obtained in human lymphoblastoid cells and skin epithelial cells following CBMN cytome assay and using flux cytometry for scoring MN [41,42,43]. However, overlapping cells, cellular debris (as observed in this study), and cytoplasmic granules can complicate the analysis by introducing confusion factors for the machine [10]. Additional cleaning steps should be incorporated during sample collection and microscope slide preparation, and adopting a standardized staining procedure may enhance the quality and reliability of nuclear abnormalities classification if we aspire to automate the method. Alternatively, artificial intelligence tools may soon become available for scoring MN in microscopic assay images [44].

Another aspect that warrants further investigation is the use of MN as stress biomarkers. Hormonal stress markers, such as cortisol, oxytocin, vasopressin, and creatinine, are recognized as non-invasive methods for quantifying animal stress [45,46,47,48,49,50,51]. Some studies show that exposure to stress and the presence of stress hormones increases DNA damage [52,53]. Conducting simultaneous measurements of MN levels and stress hormone levels would be valuable in examining the degree of correlation between these two biomarkers.

A further relevant factor concerns animal diet. It is well known that nutrition affects animal oral health, and therefore, its influence on the MN levels of oral cells cannot be disregarded [54]. This aspect requires additional exploration and should also be assessed in future studies.

Finally, the use of MN levels as biomarkers of genotoxicity has been tested in other animals and insects [14,16,30]. However, the number of studies is limited, and further research is needed to confirm the effectiveness of this biomarker as a useful tool for assessing animal welfare.

## 5. Conclusions

Our study demonstrated the feasibility of applying the BMcyt assay in dogs. Several studies in dogs employed Giemsa dye to stain nuclei, looking for DNA abnormalities [14,15,16,17,30]. We recommend the Feulgen reaction to stain the nuclei since it enables the simultaneous use of fluorescence and brightfield illumination. Double-checking cells allows the confirmation of challenging abnormalities, especially when cellular debris and cytoplasmic granules can complicate the analysis by introducing a confusion factor.

BMcyt represents an effective and minimally invasive approach that would simplify its clinical application. It is also rapid compared to cytologic and histological analysis (it can be obtained in 1–2 days), cost-effective compared to other diagnostic methods, as it only requires widely available standard equipment/reagents, and easy to execute, since it is not as technically complex as histology/pathology.

MN frequency in buccal cells has been proven to be useful as a biomarker in harmful occupational exposure of workers, lifestyle factors, and inherited genomic instability in humans [10,13,36]. However, unlike the lymphocyte CBMN assay, there is a gap in studies demonstrating that an increased MN frequency in buccal cells is prospectively associated with a higher risk of disease. More research needs to be carried out in both humans and other animals to demonstrate predictive power. We believe that this method has many advantages and great potential in veterinary practice as a biomarker of general health, and could be clinically applied to companion animals or production animals.

## Figures and Tables

**Table 1:** Key morphological and staining characteristics of the studied buccal mucosa cells that aid identification.

Buccal Mucosa Cell Type	Nuclear/Cytoplasm Ratio	Nuclear and Cell Shape and Staining	Extra Nuclei or Micronuclei
Normal Differentiated Cells	Small	Oval or round nucleus, uniformly stained. Angular and flat cell shape	No
Normal basal cells	Large	Oval or round nucleus, uniformly stained. Oval and smaller cell with darker green stained cytoplasm (brightfield illumination)	No
Cells with Micronuclei	Small	Oval or round nucleus, uniformly stained. Angular and flat cell shape	Yes (One or more micronuclei (1/3 to 1/6 the size of the main nucleus))
Binucleated Cells	Small	Oval or round nucleus, uniformly stained. Angular and flat cell shape	Yes Two nuclei with similar size
Cells with Nuclear Buds	Small	Oval or round nucleus, uniformly stained, with a constriction forming a bud. Angular and flat cell shape	No

**Table 2:** Representation of the results obtained from the counting and analysis of cells on different slides from the dogs. FB: French Bulldog; P: Pug; BN: Binucleated; NBUDS: Nuclear buds; MN: Micronuclei.

Dogs	Breed	Pregnancy	Normal Cells	Basal Cells	BN	NBUDS	MN	Total Abnormalities	Total Cells
Case 1	FB	No	881	107	6	2	3	11	1000
Case 2	FB	No	873	119	3	1	4	8	1000
Case 3	FB	Yes	903	89	0	3	5	8	1000
Case 4	FB	Yes	900	96	2	0	2	4	1000
Case 5	P	No	905	88	1	2	4	7	1000
Case 6	P	No	900	90	4	1	1	6	1000
Average	-	-	894	98.2	2.7	1.5	3.2	7.3	1000

**Table 3:** Comparative results of studies conducted in humans and dogs by various authors, including the present study. The means per 1000 cells counted are indicated.

Species	Subjects (n)	Characteristics	Subject	Basal Cells	MN	NBUD	BN	Reference
Humans	30	Youngsters	Baseline values in healthy humans	27.4	0.30	0.93	11.63	Thomas et al. 2009 [13]
	30	Elders	Baseline values in healthy humans	93.53	1.43	1.16	15.80	Thomas et al. 2009 [13]
Dogs	30	Shelter	Stress-associated genomic damage in shelter dogs	-	3	2.8	-	Santovito et al. 2022 [15]
	30	Family environment	Stress-associated genomic damage in shelter dogs	-	0.83	1.3	-	Santovito et al. 2022 [15]
	77	Pure-bred (in family environment)	Inbreeding-associated genomic damage in dogs	-	2.25	2.03	1.09	Santovito et al. 2024 [17]
	75	Mixed-bred (in family environment)	Inbreeding-associated genomic damage in dogs	-	0.71	0.99	1.33	Santovito et al. 2024 [17]
	6	Breeding females (kennel)	Baseline values in female breeding dogs	98.2	3.2	1.5	2.7	Present study

## Data Availability

Data are contained within the article.

## Appendix A

**Table A1:** Main aspects of the BMCyt assay application in animals reported in the literature.

Species	Especificities	Objective	Total Subjects (n)	Main Conclusions	Reference
Pigeons	Wild birds, and urban domestic and feral pigeons	Adaptation of the BMCyt assay for use in wild birds	245	The BMCyt assay can be used on wild birds and can assess environmental genotoxicity in pigeons, but age and sex significantly influence baseline MN frequency.	Shepherd et al. 2012 [13]
Dogs and cats	Mixed-bred from shelter and Family environment	Physiological stress-associated genomic damage	120	There is a correlation between physiological stress conditions and increased levels of genomic damage in a sample of sheltered dogs and cats	Santovito et al. 2022 [14]
Squirrels and wild boars	Wild species in anthropized and natural environments	Adaptation of the BMCyt assay to wild mammal species	77	The buccal mucosa is a sensitive site for detecting micronuclei and other nuclear abnormalities. The BMCyt assay is a reliable tool for assessing genomic damage in wild species inhabiting both anthropized and natural environments.	Bertolino et al. 2023 [15]
Dogs	Purebred and mixed-bred from shelters	Inbreeding and genomic damage relationships	152	Inbreeding in purebred dogs may increase the levels of genomic damage	Santovito et al. 2024 [16]
